# Utilizing the Effective Xanthophyll Cycle for Blooming of *Ochromonas smithii* and *O. itoi* (Chrysophyceae) on the Snow Surface

**DOI:** 10.1371/journal.pone.0014690

**Published:** 2011-02-23

**Authors:** Yukiko Tanabe, Tomofumi Shitara, Yasuhiro Kashino, Yoshiaki Hara, Sakae Kudoh

**Affiliations:** 1 National Institute of Polar Research, Tachikawa, Japan; 2 Graduate School of Science and Engineering, Yamagata University, Yamagata, Japan; 3 Graduate School of Life Science, University of Hyogo, Akou-gun, Japan; 4 Department of Biology, Faculty of Science, Yamagata University, Yamagata, Japan; 5 Department of Polar Science, The Graduate University for Advanced Studies (SOKENDAI), Tachikawa, Tokyo, Japan; Purdue University, United States of America

## Abstract

Snow algae inhabit unique environments such as alpine and high latitudes, and can grow and bloom with visualizing on snow or glacier during spring-summer. The chrysophytes *Ochromonas smithii* and *Ochromonas itoi* are dominant in yellow-colored snow patches in mountainous heavy snow areas from late May to early June. It is considered to be effective utilizing the xanthophyll cycle and holding sunscreen pigments as protective system for snow algae blooming in the vulnerable environment such as low temperature and nutrients, and strong light, however the study on the photoprotection of chrysophytes snow algae has not been shown. To dissolve how the chrysophytes snow algae can grow and bloom under such an extreme environment, we studied with the object of light which is one point of significance to this problem. We collected the yellow snows and measured photosynthetically active radiation at Mt. Gassan in May 2008 when the bloom occurred, then tried to establish unialgal cultures of *O. smithii* and *O. itoi*, and examined their photosynthetic properties by a PAM chlorophyll fluorometer and analyzed the pigment compositions before and after illumination with high-light intensities to investigate the working xanthophyll cycle. This experimental study using unialgal cultures revealed that both *O. smithii* and *O. itoi* utilize only the efficient violaxanthin cycle for photoprotection as a dissipation system of surplus energy under prolonged high-light stress, although they possess chlorophyll *c* with diadinoxanthin.

## Introduction

Oxygenic phototrophs born and living in an aquatic environment had to evolve efficient systems for trapping light because light attenuates drastically and the wavelength distribution altered with depth in the water column. However, on land, green plants faced the problem of dealing with very strong light [1]. If photosystem II (PSII) over works with such strong light, excess active oxygen is produced which causes damage to the photochemical apparatus (photoinhibition) [2]–[5]. Thus, to live and survive in a terrestrial environment, green plants had to evolve additional countermeasures against fluctuating light intensities. In low light (LL), it is advantageous to collect photons as efficiently as possible; however, when light intensities become supersaturating for photosynthesis, phototrophs need to protect themselves from potential damage due to excess energy absorption. Antenna carotenoids play important roles in both situations [6]–[8], i.e., in light harvesting as well as in photoprotection.

**Figure 1:**
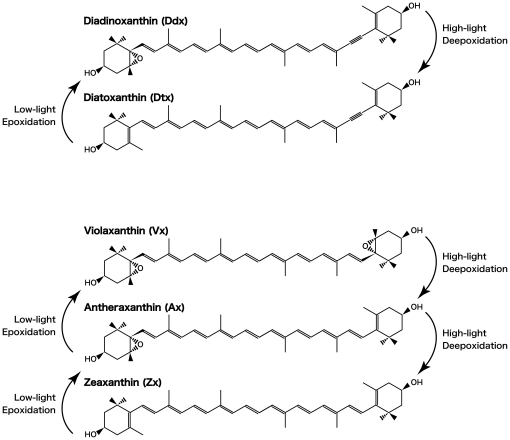
Molecular structures of the xanthophyll cycle pigments mentioned in the text. Arrows between pigments denote enzymatic conversions caused by xanthophyll cycling.

It has been generally recognized that photosynthetic organisms using the two major xanthophyll cycles to regulate dissipation of surplus light energy [9], [10] on a short time scale [11], [12] utilize either the violaxanthin (Vx) cycle, a reversible conversion of Vx, antheraxanthin (Ax), and zeaxanthin (Zx) in higher plants and green algae [13], [14] or the diadinoxanthin (Ddx) cycle, a conversion of Ddx and diatoxanthin (Dtx), in some chlorophyll (Chl.) *a*/*c*-containing algae such as diatoms, dinophytes, and haptophytes [15–17; Fig. 1]. Although the Vx cycle comprises two deepoxidation steps, the Ddx cycle involves a single step because only one of the ionone rings of Ddx carries an epoxide group (Fig. 1).

Snow algae inhabit unique environments such as alpine and high latitudes, they are especially well known in Europe, North America, Japan, the Arctic as well as Antarctica and the surrounding islands [18]–[24]. During the 20th century, many studies have been conducted on these algae to identify the different species growing on or in snow and describe the species responsible for red, green, yellow, orange, and gray snow (a comprehensive summary of the current state of knowledge of systematics, occurrence, and physiology of snow algae is given by Hoham & Duval [25]). Algae belonging to the families Cyanophyta, Chlorophyta, Euglenophyta, Chrysophyta, Pyrhophyta, and Cryptophyta have been found in snow; however, the population sizes of snow algae are best known for Chlorophyta (i.e., *Chlamydomonas* and *Chloromonas*), which color the snow green, red, and orange [26], [27].

**Figure 2:**
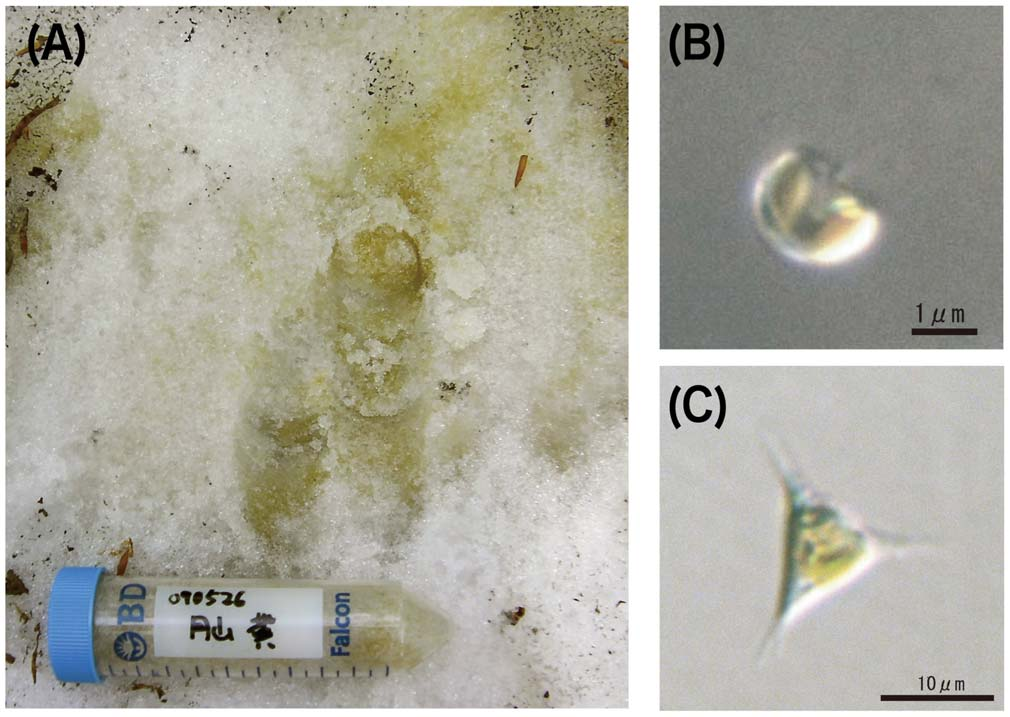
Microscopy photographs of A, *Ochromonas itoi*; B, *Ochromonas smithii*; C, landscape of yellow snow caused by *Ochromonas itoi* and *Ochromonas smithii* on/in the deposited snow surface in Mt. Gassan.

The chrysophytes *Ochromonas smithii* and *Ochromonas itoi* dominate in yellow-colored snow patches (Fig. 2) and are frequently encountered in heavy snow-affected mountainous areas facing the Japan Sea, from late May to early July, even though the areas are less than 1000 m above sea level. These algae bloom, and make the snow color deeply and visualize within short snowmelt season in which the snow still remain and solar irradiance reaching the snow surface is the highest time of the year. Not only the *O. smithii* and *O. itoi* but also all snow algae can live and bloom with visualizing on snow or glacier during spring-summer, although they are subject to extremes in terms of cold temperatures, low nutrient availability, and high solar irradiance levels [28], [29]. In such low temperature and nutrients condition, photosynthetic productivity are generally limited by light [30], and also phototrophs are susceptible to photoinhibition. While the existence and growth of snow algae are still an enigma during snowless season [31], it is highly probable that they are dormant over the period from the data of their growth temperature zone [32]. Then it has a critical implication to proliferate and have an ability increasing the population in a very limited snowmelt time. It is considered to be quite effective utilizing the xanthophyll cycle and holding sunscreen pigments as protective system for snow algae in the vulnerable environment by light such as low temperature and strong light. Snow chlorophytes comprising a majority of snow algae all over the world, are considered to be able to regulate strong light by utilizing Vx cycle, however there has been poor previous studies experimentally demonstrated. Moreover, there is no knowledge about photo-regulation of chrysophytes snow algae. Part of the reason that the experimental study has not been curried out before is that it is difficult to isolate and grow as unicellular cultures, and determine the photosynthetic properties for these snow algae.

A question arise how can the chrysophytes snow algae grow and bloom under such a low temperature and nutrients, and strong light environment? To dissolve this question, we studied with the object of light which is one point of significance to this problem. Therefore, we tried to establish unialgal cultures of *O. smithii* and *O. itoi*, and examined their photosynthetic properties such as non-photochemical quenching (NPQ) and the corresponding function of xanthophyll cycling before and after illumination with high-light (HL) intensities in an experiment by using the cultivated strains.

## Results and Discussion

It is considered to be quite effective utilizing the xanthophyll cycle as protective system for snow algae because they live in the vulnerable environment by light such as low temperature and strong light and have an ability increasing the population in a very limited snowmelt time. How can the chrysophytes snow algae grow and bloom under such a low temperature and nutrients, and strong light environment? To dissolve this question, it is important to study with the object of light which is one point of significance to this problem. However any experimental studies have not been curried out previously, and this was partly due to the difficulty to establish unicellular cultures and determine the photosynthetic properties for the chrysophytes snow algae. Therefore, we collected the yellow snow samples and measured photosynthetically active radiation (PAR) at Mt. Gassan, Japan when the snow algae bloomed. Microscopic observation of the snow sample was performed to determine the dominant species, and then, we tried to establish unialgal cultures of each dominant species. Using the unicellular cultures, the light irradiation experiment was done to elucidate the working of xanthophyll cycle and the corresponding xanthophyll cycle pigments. The pigment compositions were analyzed by a high performance liquid chromatography (HPLC) after cessation the de-epoxidase activity in the xanthophyll cycle (Fig. 1), and their photosynthetic responses such as maximum quantum yield of PSII, relative electron transport rate (rETR), and NPQ using a pulse amplitude modulation (PAM) chlorophyll fluorometer, to make sure the working of xanthophyll cycle and which pigments are utilizing in these chrysophytes snow algae before and after illumination with HL intensities (1500 µmol/m^2^/s) by using the cultivated strains.

Yellow snow algal community collected at Mt. Gassan in May 2008 was dominated by two freshwater chrysophytes, *O. smithii* and *O. itoi*, which form extensive colorations of snow (Fig. 2). While the temperature on the snow surface was at about 0°C, such the cold environment is not enough to perform photosynthesis for the common algae. However the two species can grow up at 0°C and this means that they are adapted to cold environment. Unicellular cultures of *O. smithii* and *O. itoi* were established aseptically for the first time ever, at 4°C in AF-6 medium with 10 µmol/m^2^/s of PAR. *O. itoi* is 2–3 µm diameter of spherical or piriform cell, and there is one discotic chloroplast and no cell wall, they have two anisometric flagella (Fig. 2B). *O. smithii* has about 10 µm diameter of cell, and spiny projections, and a discotic chloroplast, non cell wall, and two anisometric flagella as *O. itoi*. Although there are one or two spiny projection(s) on the cell surface and it is similar to tetrapod-like form in morphology (Fig. 2C), the morphology transformation is observed from tetrapod to non-spiny spherical form under the cultural condition. Both two species were observed that the cells explode under room temperature.

**Figure 3:**
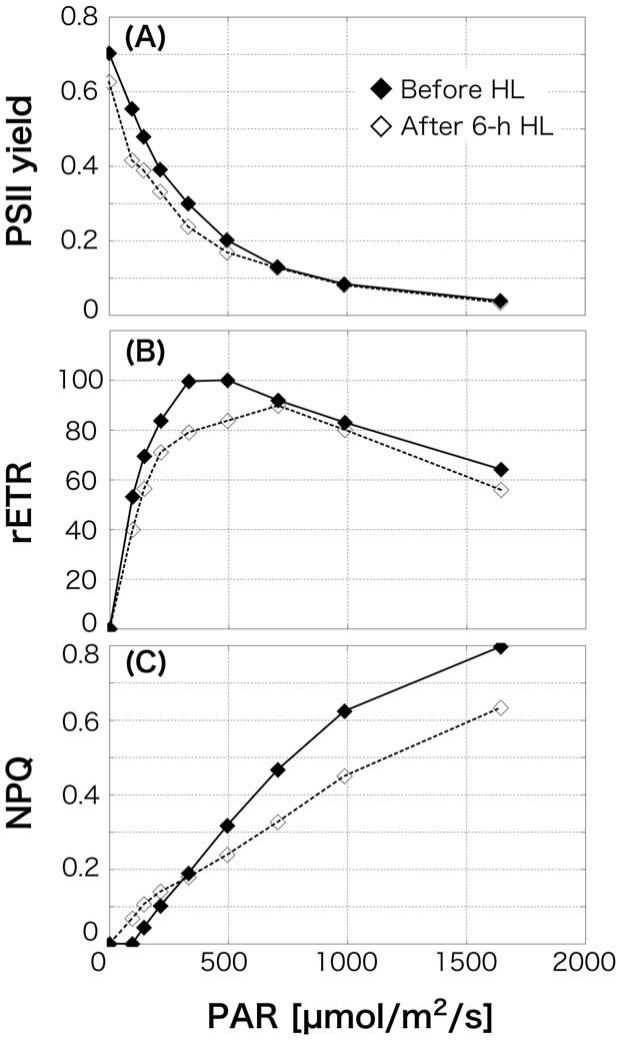
Change in photosynthetic responses of *Ochromonas itoi* determined by a PAM fluorometer before illumination and after 6-h high-light (HL) illumination. A, Relationship between photosynthetically active radiation (PAR) and photosystem II (PSII) yield; B, relationship between PAR and relative electron transport rate (rETR); C, relationship between PAR and non photochemical quenching (NPQ).

Using a PAM chlorophyll fluorometer, chlorophyll fluorescence binding to PSII were measured and PSII yield, rETR, and NPQ at various light intensities were determined. Chlorophyll fluorescence indicates the energy destination after the absorption into the chlorophylls, such as photochemical reaction at PSII, heat, fluorescence, and photochemical reaction at photosystem I (PSI). Maximum quantum yield of PSII (PSII yields under no actinic light which was obtained from dark adapted cells, indicating the stress condition) of *O. itoi* was 0.70 (Fig. 3A) under LL (10 µmol/m^2^/s). This indicates that they were not subject to stress, compared with the healthy non-stressed yield of benthic algae and phytoplankton (0.65, cf. [33]). Photo-inhibition of *O. itoi* was observed at 495 µmol/m^2^/s of PAR (Fig. 3B), and NPQ indicating xanthophyll cycling was detected from 145 µmol/m^2^/s of PAR (Fig. 3C) in the LL condition. After 6-h HL irradiance, there was slight decrease in the maximum yield of PSII to 0.63. Relative electron transport rate also declined. NPQ was detected at 96 µmol/m^2^/s of PAR, and the value at a high PAR range (<332 µmol/m^2^/s) was lower than that before illumination with HL. Clearly observed NPQ in both algae despite a 10 µmol/m^2^/s LL condition, shows that they intrinsically provide a highly functional heat dissipation system by xanthophyll cycling, and the detected NPQ at lower PAR after HL treatment suggests that they could obtain higher ability of xanthophyll cycling with response to the strong light than under LL (Fig. 3C). Because of this investment in photo-protection, the photosynthetically light use efficiency may have led to be lower throughout the whole light intensities under HL condition.

From the pigments analysis using a HPLC, both *O. itoi* and *O. smithii* possessed pigments not only typical of those found in chrysophytes, Chl.*a* and *c*, and the primary carotenoids, α- and β-Car, Fx, and Ddx as xanthophyll cycle pigments, but also, in addition, a series of Vx cycle pigments, Vx, Ax, and Zx in LL before HL illumination (Table 1). This results suggested that the chrysophytes snow algae dissipate surplus light energy using both xanthophyll cycles, as reported in a previous study showing that algae with Chl.*a/c* display the Ddx and Vx cycles [34].

**Table 1:** Pigment composition of *Ochromonas itoi* and *Ochromonas smithii* before high light (HL) and after 6-h HL incubation.

	Pigments [mol/100 mol Chl.*a*]								
	Chl.*a*	* ^1^ Chl.*c*	Fx	Ddx	Dtx	Vx	Ax	Zx	* ^2^ α+β-Car
Before HL *O. itoi*	100	16.1	55.4	0.650	0	15.7	0.293	0.212	20.5 (α+β)
*s.d.*	-	0.0968	0.397	0.0255	-	0.288	0.0158	0.00387	0.304
After 6-h HL *O. itoi*	100	27.1	64.6	1.06	0	19.0	1.12	0.504	26.8
*s.d.*	-	0.0146	0.0204	0.0234	-	0.108	0.00711	0.00327	0.300
Before HL *O. smithii*	100	17.3	58.3	0.378	0	14.1	0.322	0.427	6.29 (β)
*s.d.*	0	0.0361	0.155	0.0234	-	0.108	0.0174	0.00341	0.0214
After 6-h HL *O. smithii*	100	17.3	57.5	0.386	0	13.3	0.979	0.835	6.19 (β)
*s.d.*	0	0.00474	0.0184	0.000462	-	0.105	0.0240	0.00258	0.0256

Chl.*a*, chlorophyll *a*; Chl.*c*, chlorophyll *c*; Fx, fucoxanthin; Ddx, diadinoxanthin; Dtx, diatoxanthin; Vx, violaxanthin; Ax, antheraxanthin; Zx, zeaxanthin; α-Car, α-carotene; β-Car, β-carotene. Values are means of three independent measurements, and s.d. are standard deviation.

*^1^ Chl.*c*, Chl.*c*1+*c*2

*^2^ α-Car and β-Car were not absolutely separated using this study's HPLC method. α-Car was only detected in *O. itoi*.

**Figure 4:**
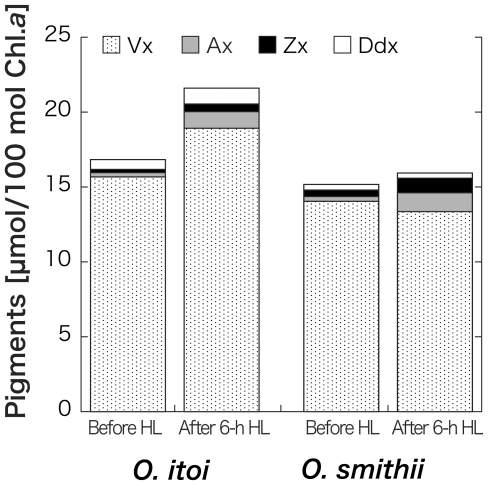
Changes in xanthophyll cycle pigments in *Ochromonas itoi* and *Ochromonas smithii* after 6-h high-light (HL) incubation.

**Figure 5:**
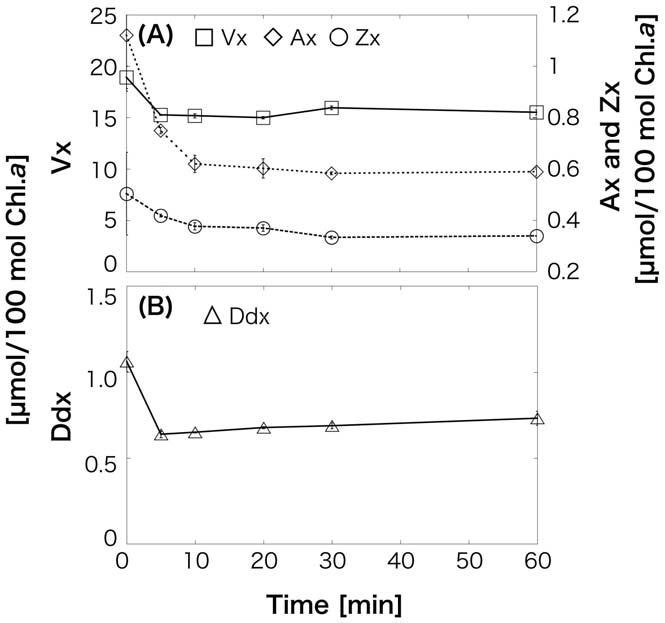
Time course of xanthophyll cycle pigment changes in a cell suspension of *Ochromonas itoi* in low-light (LL) during 1-h illumination after 6-h high-light (HL) illumination. Pigments are normalized to chlorophyll *a* (Chl.*a*). Values are means of three independent records, and error bars are standard deviations. A, Epoxidation of zeaxanthin (Zx) from antheraxanthin (Ax) to Vx; B, epoxidation of diatoxanthin (Dtx) to diadinoxanthin (Ddx).

**Figure 6:**
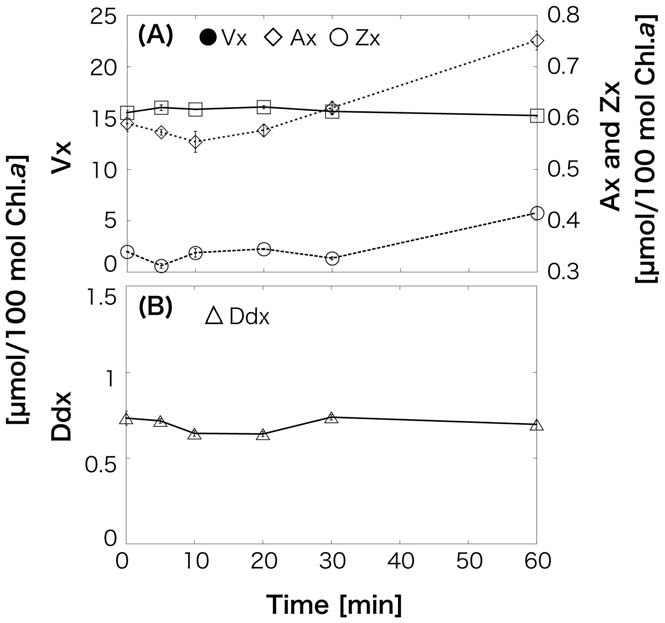
Time course of xanthophyll cycle pigment changes in *Ochromonas itoi* in high-light (HL) after 6-h HL illumination, followed by 1-h low-light (LL) illumination. Pigments are normalized to chlorophyll *a* (Chl.*a*). Values are means of three independent records, and error bars are standard deviations. A, Deepoxidation of violaxanthin (Vx) from antheraxanthin (Ax) to zeaxanthin (Zx); B, deepoxidation of diadinoxanthin (Ddx) to diatoxanthin (Dtx).

After 6-h HL irradiation, all four pigments increased in *O. itoi* (Vx: 15.7 (±0.288)–18.9 (±0.108); Ax: 0.293 (±0.0158)–1.12 (±0.00711); Zx: 0.212 (±0.00387)–0.504 (±0.00327); Ddx: 0.650 (±0.0255)–1.06 (±0.0234); Fig. 4). Decrease in Ddx and Vx from 0.378 (±0.0234) mol/100 mol Chl.*a* to 0.353 (±0.000462) mol/100 mol and from 14.1 (±0.108) mol/100 mol to 13.4 (±0.105) mol/100 mol, respectively, and increase in Ax and Zx from 0.322 (±0.0174) mol/100 mol to 1.26 (±0.0240) mol/100 mol and 0.427 (±0.00341) mol/100 mol to 0.971 (±0.00258) mol/100 mol, respectively, were observed in *O. smithii*. Although the pool size of Vx cycle pigments (Vx+Ax+Zx) increased in both species, Dtx was not detected. The values are means of three independent measurements, and error estimates in parenthesis are standard deviations. This increase of the Vx cycle pool size supports to the photosynthesis data in which showed the detected NPQ and the decrease of light use efficiency at lower PAR after 6-h HL irradiation. In LL illumination after 6-h HL, Vx, Ax, and Zx in *O. itoi* began to decrease after 5 min, and Ax and Zx then continued to decrease until 30 min (Fig. 5A). Ddx also decreased at 5 min; however, Dtx was never detected (Fig. 5B). Then, Vx and Ddx did not almost change during 1-h HL after 6-h HL followed by 1-h LL, but Ax and Zx gradually increased for 1 h (Fig. 6). These results showed that the deepoxidation reactions from Vx to Ax and Zx and the epoxidation reactions from Ax and Zx to Vx occur under HL and LL, respectively. Although the two snow algae were thought to utilize both Ddx and Vx cycles as xanthophyll cycles at first, the pigments analysis after HL and the time-course irradiation experiment confirmed that only the Vx cycle operates in both snow algae, even though they possess Ddx. The molar ratios of Ddx/Chl.*a* shown in Table 1 clearly indicate that the amount of Ddx is too low to be functional in photoprotection.

Chlorophyta are one of the most evolutionarily ancient eukaryotic alga which is considered to have been born by a primary endosymbiosis of a photosynthetic cyanobacterial-like prokaryote inside a eukaryotic phagotroph [35], and fossils have been found from Precambrian times. Chlorophyta originated in the ocean; they then succeeded in colonizing land after completion of the ozone layer. Coping with strong light was a big problem for them in the terrestrial environment. They consequently developed a system to avoid damage to the photochemical apparatus for dissipating excess light energy as heat by production and decomposition reactions called the xanthophyll cycle with Vx-Ax-Zx. According to a generally accepted theory, Chl.*a*/*c*-containing algae evolved from red algae, which are also one of the most ancient eukaryotic alga, by a secondary endosymbiosis over a long natural history of oxygenic phototrophs [35]. Almost Chl.*a*/*c*-containing algae are thought to possess a Ddx cycle as the xanthophyll cycle in place of the Vx cycle [15], but some Chl.*a*/*c*-containing algae are known to hold either the Vx cycle or Ddx cycle [36]–[37]. Both Ax and Dtx have 10 conjugated double bonds; however, the degree of conjugation is thought be higher in Ax than Dtx because the terminal conjugated double bond of Dtx is located at some distance from the next one (Fig. 1). Therefore, the quenching ability is higher in Ax because there are two reaction steps in the Zx cycle, and the energy gap from Chl.*a* is larger in Ax with its lower energy level of singlet excited states (S_1_) than Dtx [38]. Therefore, the Ddx cycle as a system that regulates a small amount of light energy is considered suitable for almost Chl.*a*/*c*-containing algae that live and have evolved under water being low-intensity light environment. Vx is rarely seen in chrysophytes, but diatoms which bloom in spring in ocean and possess the same Chl.*a*/*c* as chrysophytes prevail utilizing the Ddx cycle as dissipation system [39]–[42]. Considering this knowledge and the results of this study, it seems that Chl.*a*/*c*-containing algae develop the xanthophyll cycle depending on their each habitat.

This study's algae grow and bloom on snow deposits on beech forest (deciduous forest) floors in early spring (mid-May to early-June). This is a unique light environment because the intensity of sunlight fluctuates drastically due to sudden direct light shining through the young leaf canopy combined with reflection and scattering of sunlight on the snow surface. In fact, PAR value at noon during the blooming period, measured by a spherical sensor, immediately changed from 1842 µmol/m^2^/s to 3843 µmol/m^2^/s in less than 5 min from 11:55 am to 12:05 pm on 26 May. It is quite significant to utilize the xanthophyll cycle as one of protective system for snow algae to live and bloom in the vulnerable environment by light under low temperature during a very limited snowmelt time. However any experimental studies on snow algal xanthophyll cycling have not been curried out previously, especially for chrysophytes snow algae. In this study, we have succeeded to establish the world's first unialgal cultures of two dominant snow algae in the yellow snow. Using the unicellular cultures, our experimental study demonstrated that the chrysophytes snow algae have high photoprotection ability, and in addition, they perform it by utilizing not Ddx cycle, but only efficient Vx cycle under strong light. The Ddx cycle which almost Chl.*a/c*-containing algae utilized may be unnecessary for the snow algae because their extreme habitat, and this may be the reason for populations of snow algae being dominated by chlorophyta [28], [29] definitely possessing this effective Vx-Ax-Zx system. Moreover, the chrysophytes snow algae could have survived and bloomed along the course of evolution with adapting to this unique snow environment by acquiring the high regulation system against light.

## Materials and Methods

### Algal strains and culture conditions

The chrysophytes *O. smithii* and *O. itoi* were sampled from snow deposited on Mt. Gassan (38°30′N, 139°60′E), Yamagata Prefecture, Japan, in May 2008. Unicellular cultures were established aseptically using AF-6 medium (cf. Microbial Culture Collection, National Institute for Environmental Studies, Japan; http://mcc.nies.go.jp/02medium-e.htmljsessionid=23C606E1056F4DC40E66FF01A0F561AE#AF-6) at 4°C in 500 mL conical flasks with continuous LL illumination of 10 µmol/m^2^/s PAR, as measured in the empty culture vessels. Chl.*a* concentrations of both algae were kept at 1–2 mg/L, as determined by fluorescence measurements. At this density, all cultures were colored yellow or gold.

### Light irradiation experiment

The cultures were exposed to 6-h HL with 1500 µmol/m^2^/s PAR by cold light (HL-150, HOYA) with gentle stirring at 4°C in a temperature-controlled chamber, followed by 24-h LL to acclimation before the experiments started. After 6-h HL exposure, three sets of time-course experiments were conducted as follows. The cultures were returned to the LL condition for 1 h, and then 10- and 2-mL aliquots from the cultures were sampled at 5-min intervals for pigment analysis and photosynthesis measurements, respectively. Immediately after sampling for pigment analysis, dithiothreitol was added at a final concentration of 300 µM to the sampled cells to stop the de-epoxidase activity in the xanthophyll cycle [40]–[42]. Cells were then centrifuged at 15000×*g* and 4°C for 5 min, and the precipitate was freeze-dried. The samples were extracted in *N,N*-dimethylformamide solution at −20°C for 20 h in darkness. After 1-h LL, each culture was again exposed to HL until 1 h, and pigment analysis and photosynthesis measurements were performed as described above.

### Measurements of photosynthesis

Photosynthetic yield (PSII quantum yield) and NPQ were measured using a Water-PAM fluorometer (Waltz) with control and analysis software, Win-control, under nine stepwise actinic light intensities (0, 96, 145, 214, 332, 495, 707, 988, 1644 µmol photons/m^2^/s of PAR with 30 s duration) and >2,000 µmol photons/m^2^/s of saturating pulse with 0.4 s duration for determination of the light photosynthetic rate (determined as relative electron transport rate, rETR) at 4°C in a temperature-controlled chamber. The gain value of photoelectric multiplier (PM-Gain) was set to 3 throughout the whole measurements. After incubation of each sample in dark conditions for 10 min, 2 mL of the sample was transferred to the measuring quartz cuvette of the fluorometer, and a stirring apparatus was installed. Light curves were obtained by running a rapid light curve protocol in Win-control software. The photosynthetic rate, expressed as rETR [43], was as follows:
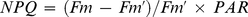
(1)where F and Fm' are the transient and maximum fluorescence levels at certain actinic light intensities at a given time. Then (Fm' − F)/Fm' indicates PSII yield, and PAR is the photosynthetically active radiation. NPQ was as follows:
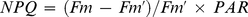
(2)where Fm is the maximum fluorescence level of non-illuminated samples.

### Pigment analysis

The extracted samples were purified using 0.20-µm, PTFE, HPLC syringe cartridge filters (DISMIC-13_JP_, ADVANTEC). Separation was achieved using a Shimadzu Prominence series HPLC (LC-20AT) with a system controller (CBM-20A), refrigerated autosampler compartment (SIL-20A), thermostatically controlled column compartment (CTO-10AS_VP_), dual pump with in-line vacuum degasser (DGU-20A), and photodiode array detector set (SPD-M20A) to monitor at wavelengths from 300 nm to 750 nm, and a Phenomenex LUNA C8(2) column (150 mm×4.6 mm; 3-µm particle size) protected by a Phenomenex guard cartridge (C8; 4×3.0 mm). The gradient elution program was performed according to the method described by Heukelem and Thomas [44] with some modifications. Solvent A was 70∶30 (v/v) methanol and 28 mM aqueous tetrabutyl ammonium acetate (pH 6.5), and Solvent B was methanol, eluted as described below at a flow rate of 1 mL/min over a period of 72 min. The ratio of Solvent B increased linearly from 20% to 45% over the first 18 min, then gradually increased to 90% until 65 min, and finally 95% at 66 min. The ratio of 95% Solvent B was kept for 5 min to elute highly hydrophobic pigments. Detected pigments were analyzed by Shimadzu CLASS-Agent Manager ver. 2.30 and LabSolution ver. 1.21 SP1.

The HPLC system was calibrated using authentic pigment standards from the DHI Institute of Water and Environment, Denmark, and the separated algal pigments were identified and quantified by comparing retention times and absorption patterns.

### PAR measurement in natural conditions

Changes in light environment were measured using a PAR logger (MDS type-L, Alec) from 10:00 am on 25 May to 9:00 am on 27 May, 2009, on Mt. Gassan (38°30′35.0″N, 139°59′50.6″E), Yamagata Prefecture, Japan. The logger has a spherical (270°) sensor, then the recorded PAR data were the sum of the direct, reflex, and scattering radiations on snow surface where the snow algae bloomed. The logger was pre-calibrated by the manufacturer, who found no significant drifts in the measurements and sampled data every 1 min. The logger was placed on the snow surface in the same place as the yellow snow blooming at the time of the study.
